# Supplementary material for: Involvement of Brn3a-positive spinal dorsal horn neurons in the transmission of visceral pain in inflammatory bowel disease model mice
Source: Front Pain Res (Lausanne). 2022 Dec 7;3:979038. doi: 10.3389/fpain.2022.979038 (PMC9768036; doi:10.3389/fpain.2022.979038)
Supplement: Supplementary file 1 [file Datasheet1.pdf]

Sup Figure 1

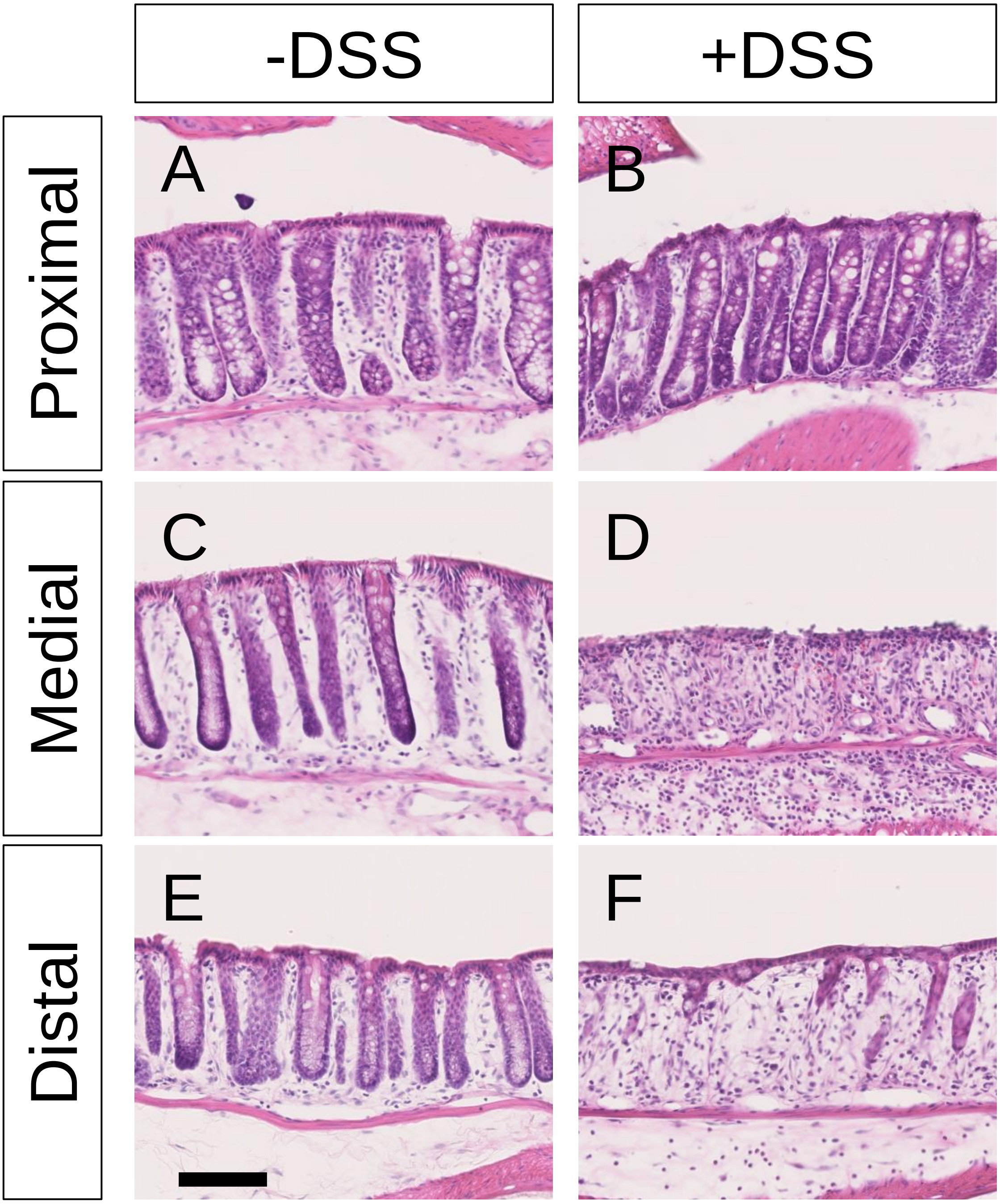

# Sup Figure 2

## A: DAI score

| Score | Weight loss | Stool | Blood |
|---|---|---|---|
| 0 | None | Normal | None |
| 1 | 0-5% | Adhere to animal bedding | |
| 2 | 5-10% | Shape became round | Slightly bleeding |
| 3 | 10-15% | Shape collapse | |
| 4 | 15-20% | Watery | Gross bleeding |

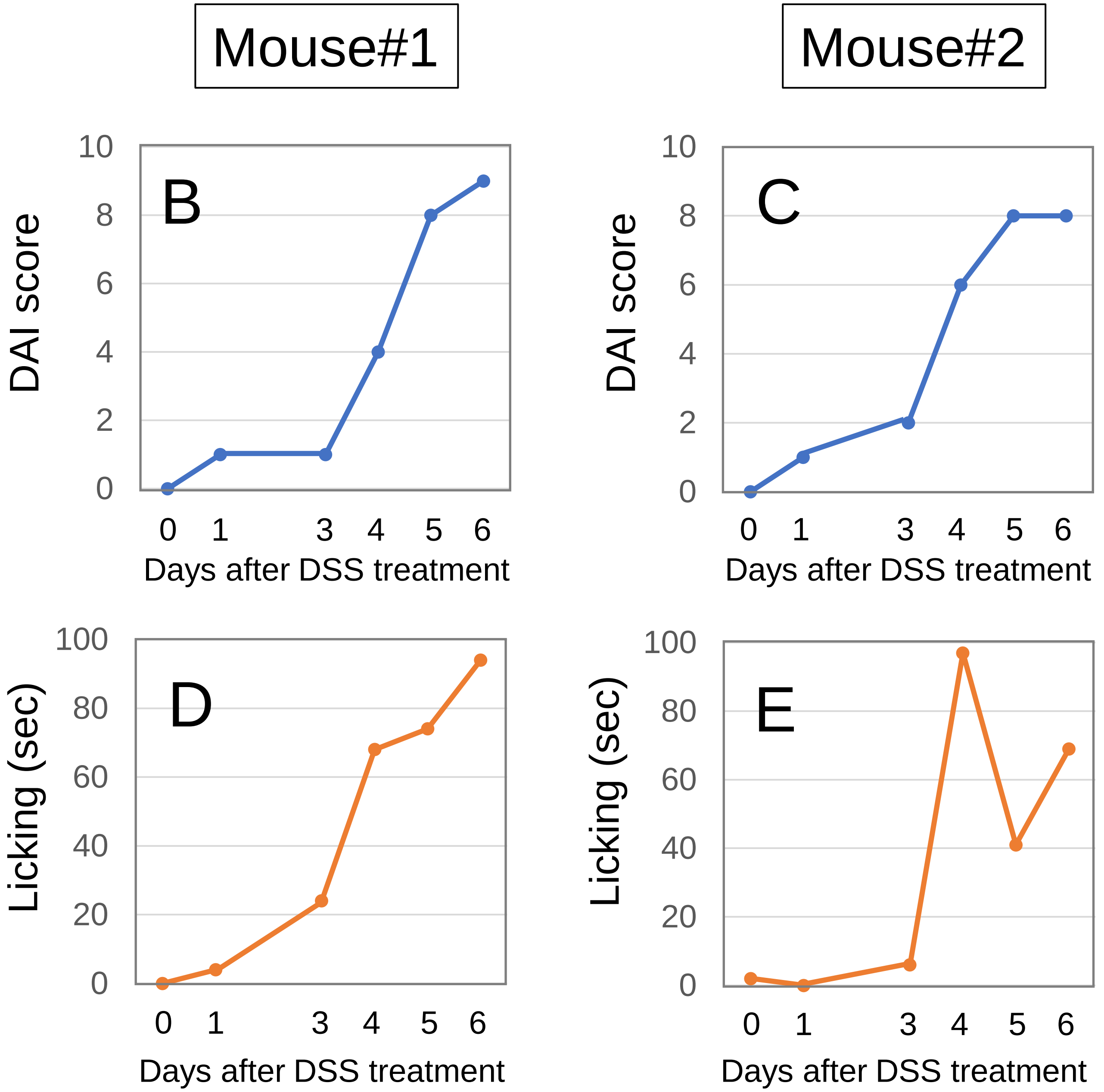

# Sup Figure 3

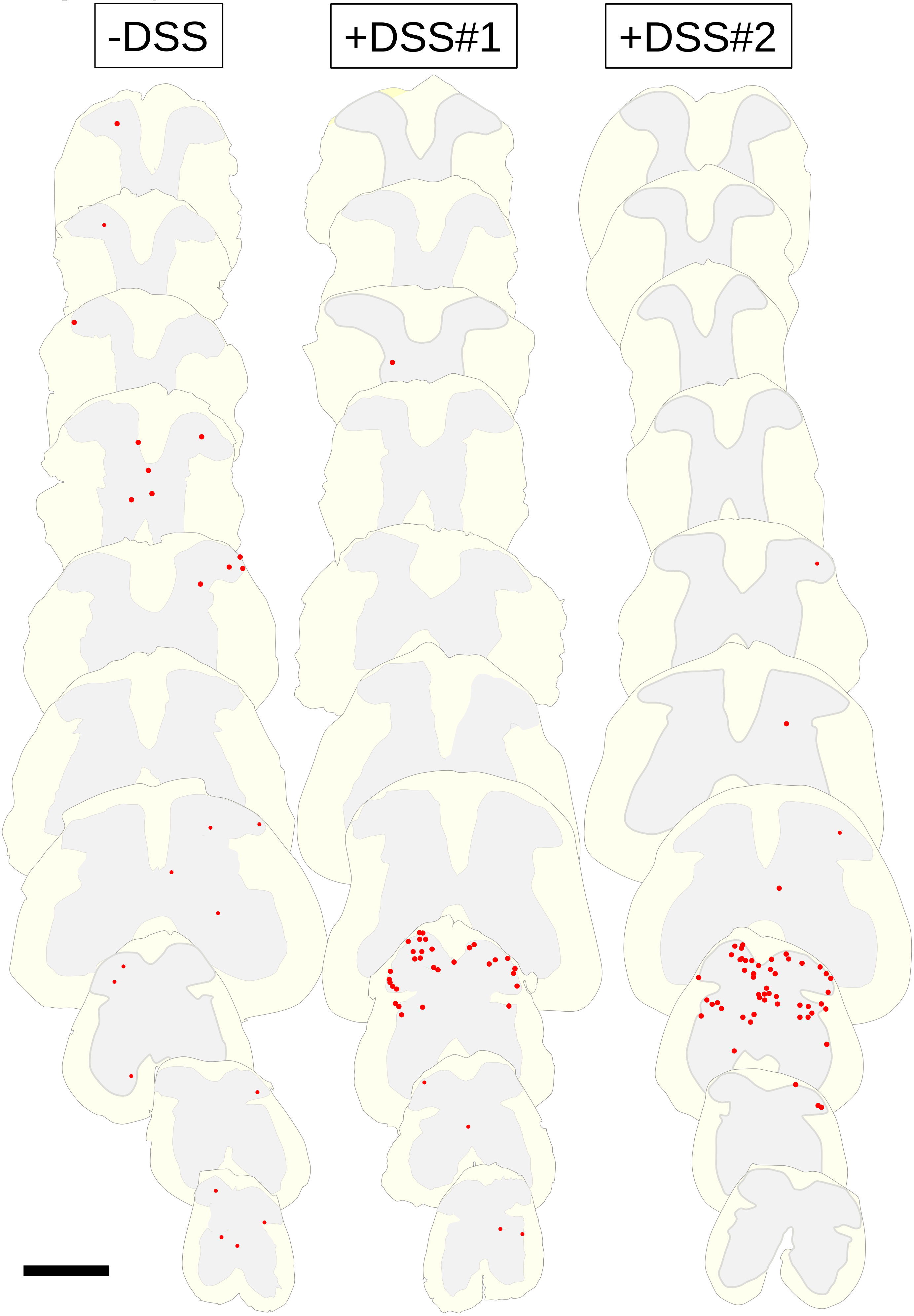

# Sup Figure 4

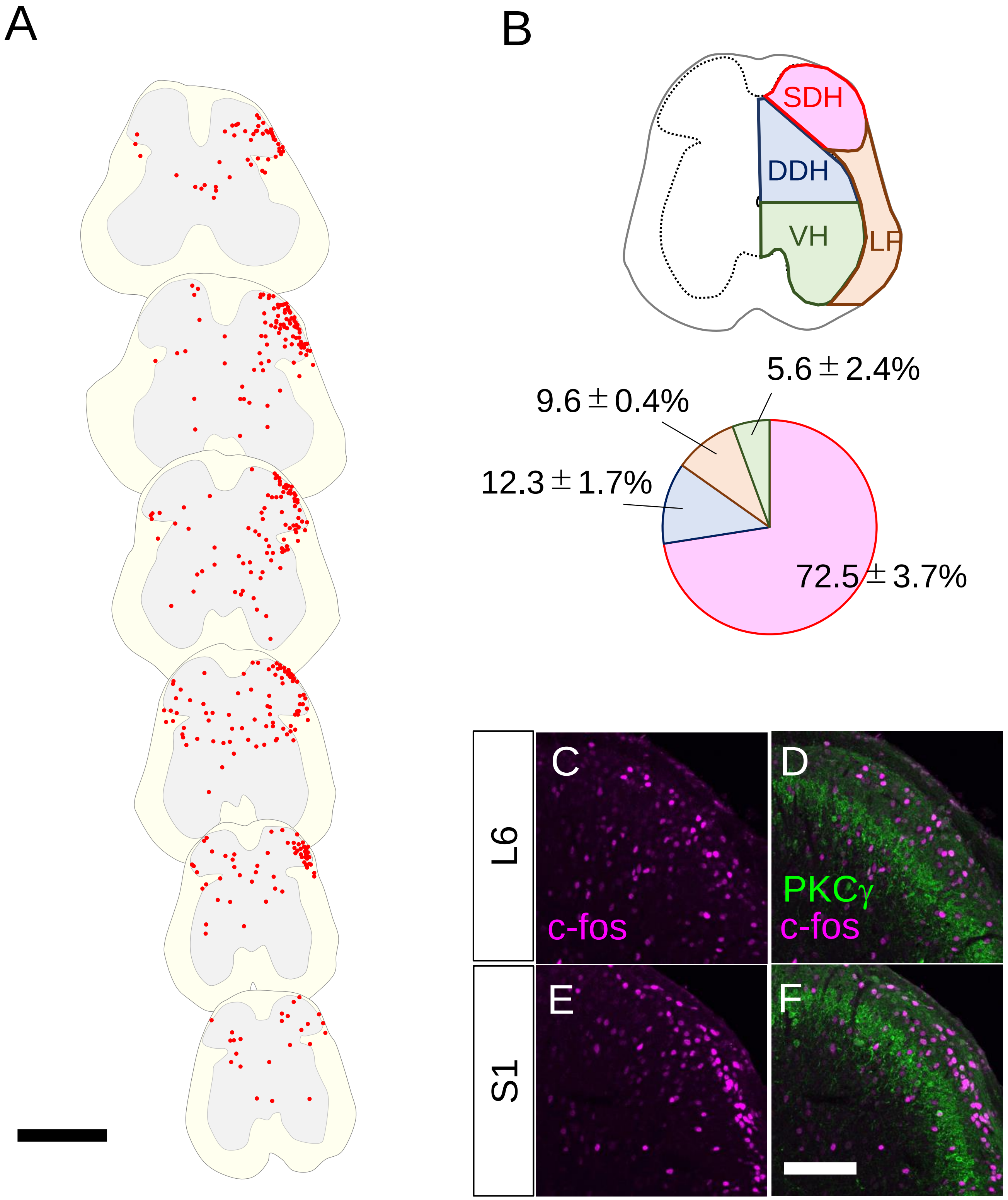
A
B
SDH
DDH
VH
LF
5.6 ± 2.4%
9.6 ± 0.4%
12.3 ± 1.7%
72.5 ± 3.7%
L6
S1
C
D
E
F
c-fos
PKCγ
c-fos

# Sup Figure 5

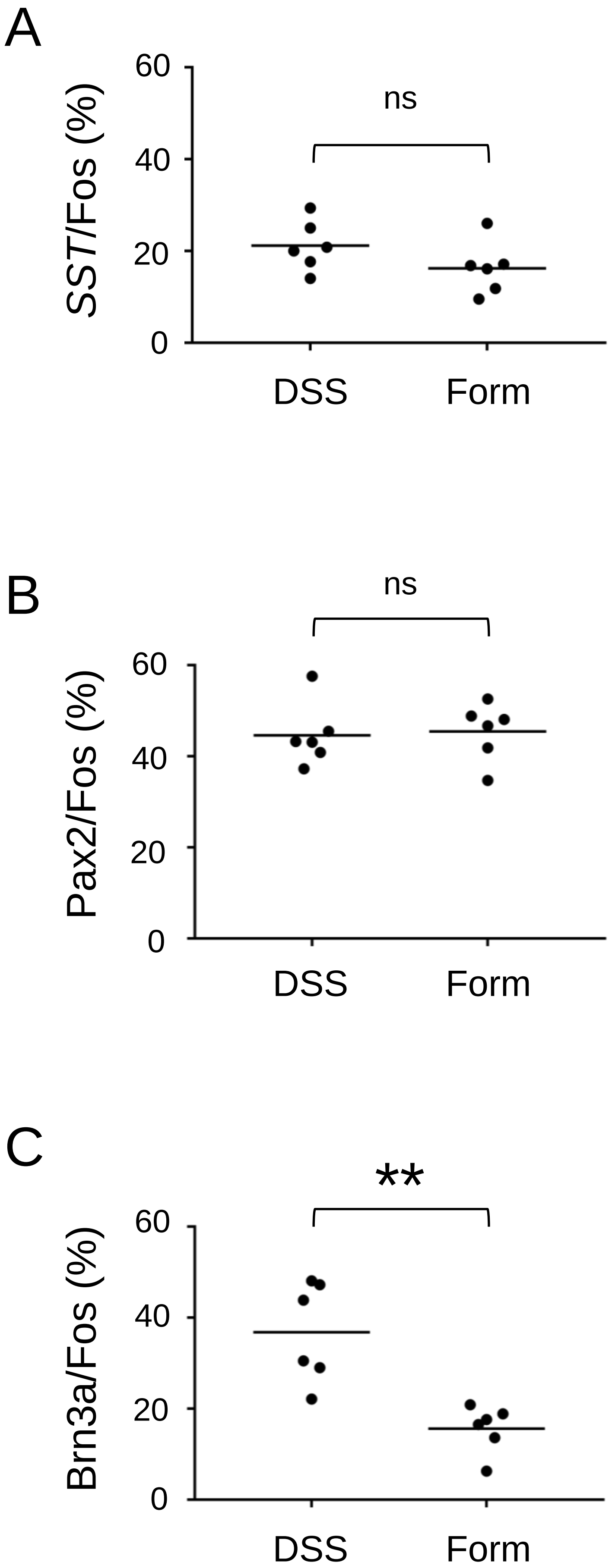
A
60
40
20
0
*SST*/Fos (%)
ns
DSS
Form
B
60
40
20
0
Pax2/Fos (%)
ns
DSS
Form
C
60
40
20
0
Brn3a/Fos (%)
**
DSS
Form

# Sup Figure 6

CTB → NTS

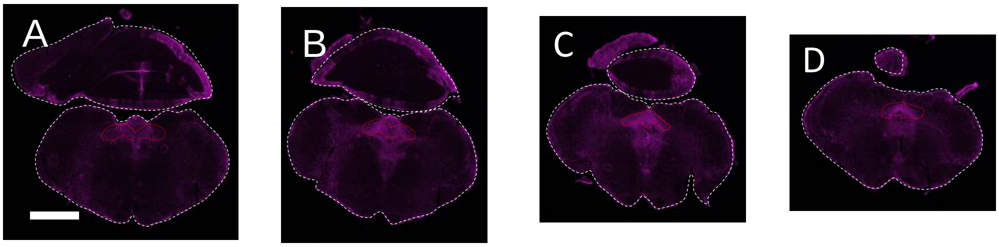


240 μm

CTB → LPb

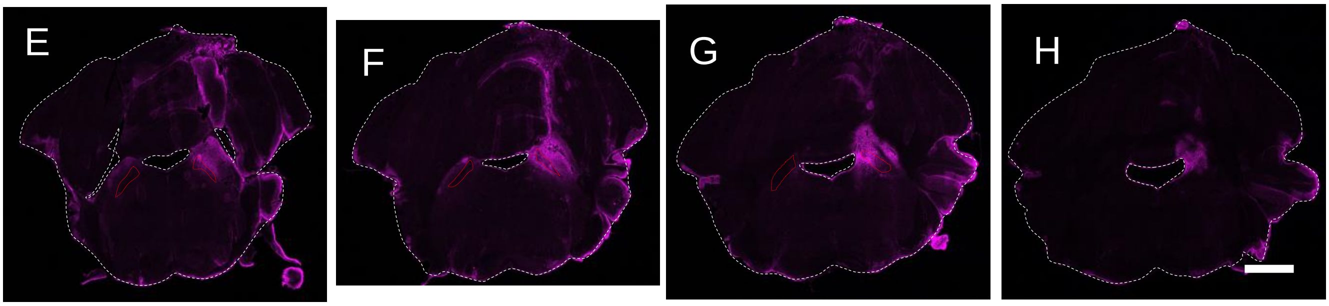


160 μm

# Sup Figure 7

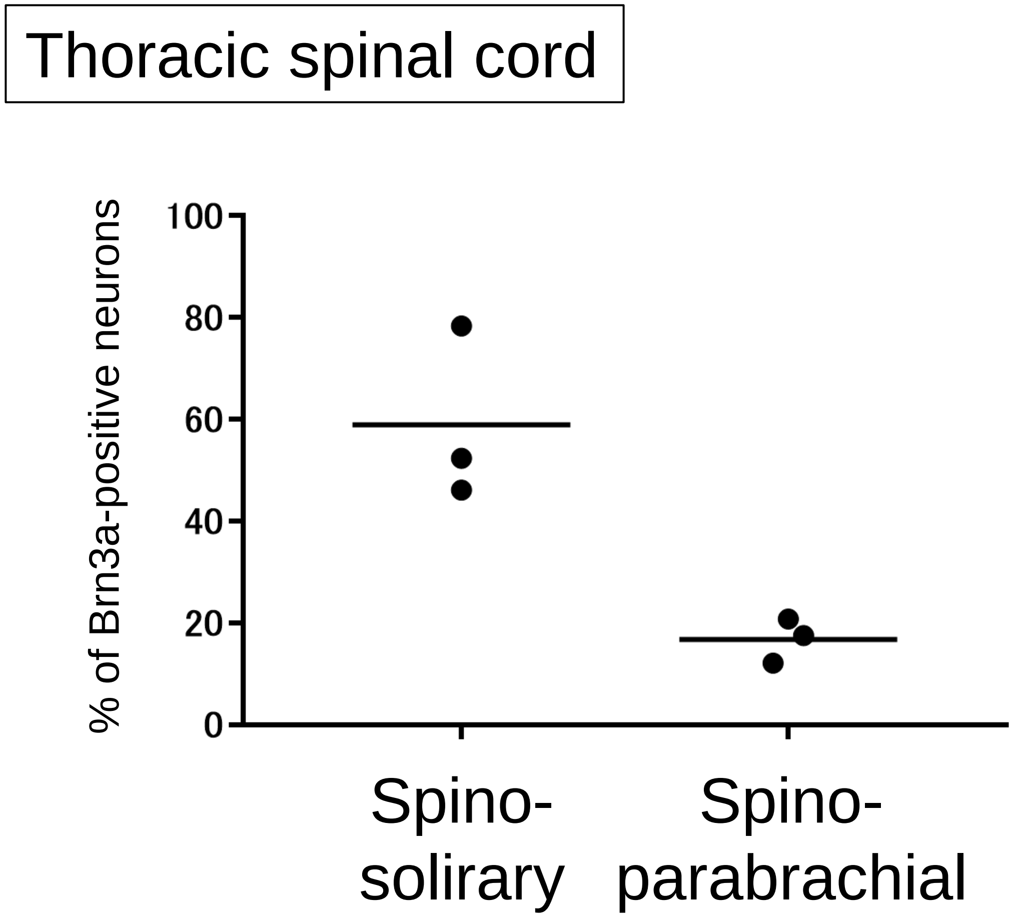

Thoracic spinal cord
% of Brn3a-positive neurons
100
80
60
40
20
0
Spino-solirary
Spino-parabrachial

# Sup Figure 8

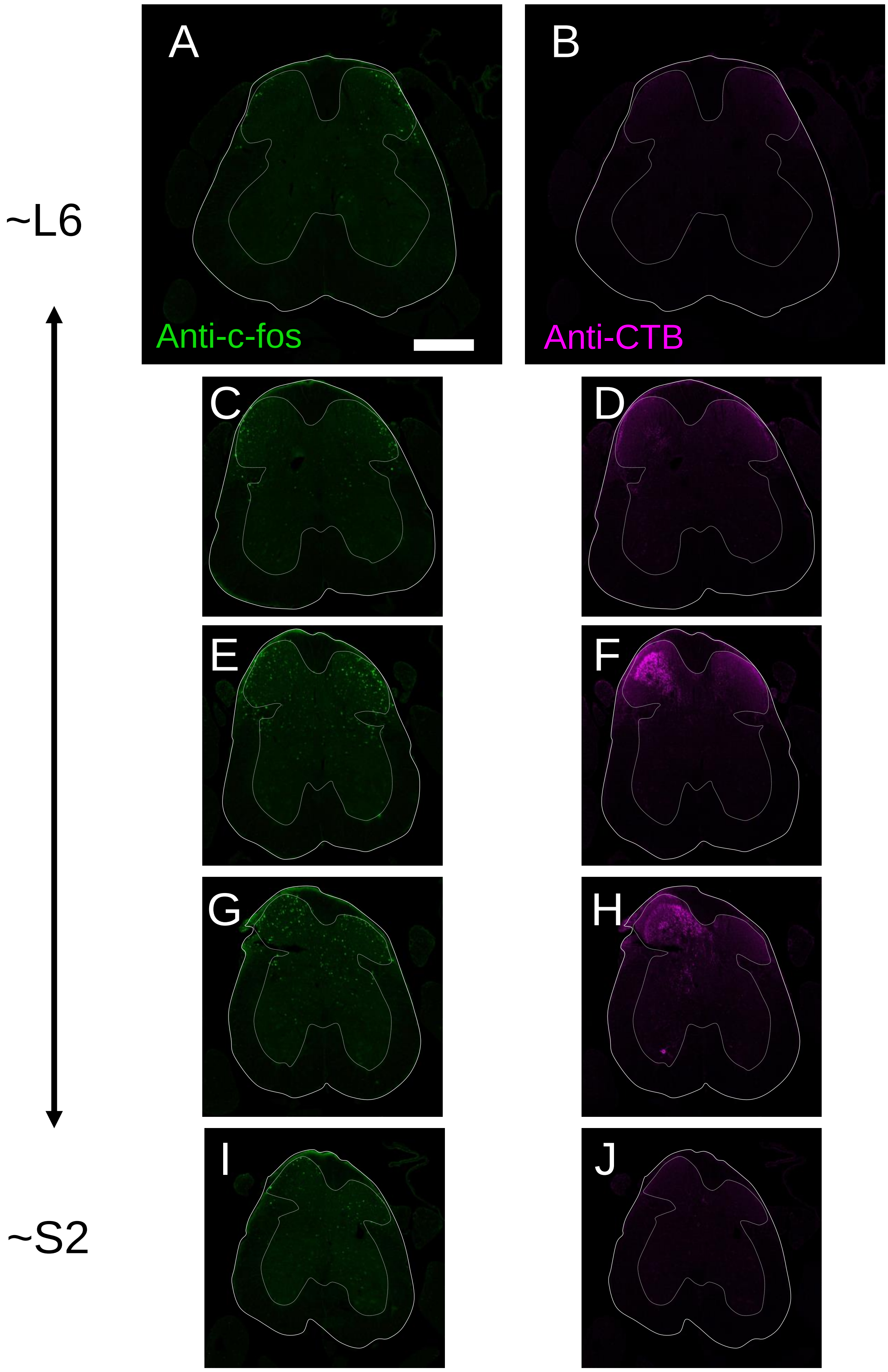

# Sup Figure 9

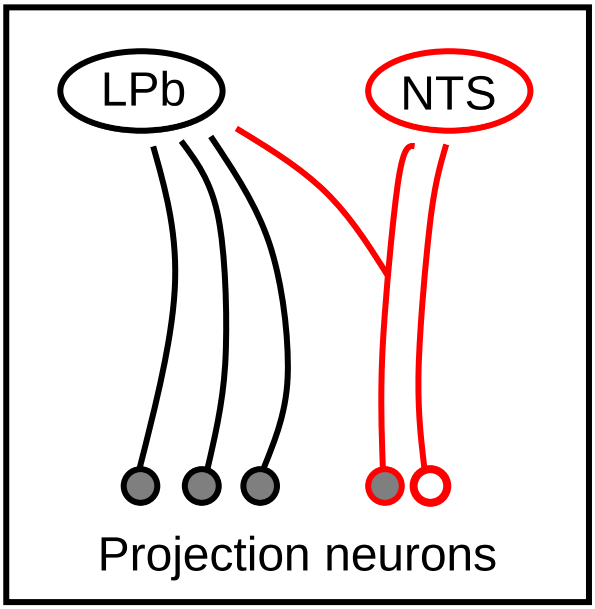


Polgar et al (2010) showed that ……

ratio of LPb projection neurons ( + ) to NTS projection neurons ( + ) is 4:1

ratio of LPb+NTS projection neurons ( ) to NTS only projection neurons ( ) is 9:1

The current study showed that ……

percentage of Brn3a positive neurons among LPb projection neurons ( + ) is 16.0%

percentage of Brn3a positive neurons among NTS projection neurons ( + ) is 51.8%

If the number of NTS projection neurons ( + ) is N

the number of LPb projection neurons ( + ) is 4N,
and the number of Brn3a-positive LPb projection neurons is <u>4N x 0.160</u>

The number of LPb+NTS projection neurons ( ) is 0.9N,
and the number of Brn3a-positive LPb+NTS projection neurons is <u>0.9N x 0.518</u>

The number of LPb only projection neurons ( ) is 4N – 0.9N = <u>3.1N</u>,
and the number of Brn3a-positive LPb only projection neurons is <u>4N x 0.160 – 0.9N x 0.518</u>

Taken together, the percentage of Brn3a-positive neurons among LPb only projection neurons is

$$\frac{4N \times 0.160 - 0.9N \times 0.518}{3.1N} \times 100 = 5.61\%$$
