# Supplementary material for: Involvement of Brn3a-positive spinal dorsal horn neurons in the transmission of visceral pain in inflammatory bowel disease model mice
Source: Front Pain Res (Lausanne). 2022 Dec 7;3:979038. doi: 10.3389/fpain.2022.979038 (PMC9768036; doi:10.3389/fpain.2022.979038)
Supplement: Supplementary file 3 [file Datasheet3.pdf]

Supplementary Table 1. The number of Brn3a-positive neurons among c-fos-positive neurons. The number of Brn3a-positive neurons among c-fos-positive neurons in 6 DSS-treated and 6 formalin-injected mice described in Figure 4C is shown.

	number of Brn3a-positive neurons	number of c-fos-positive neurons
DSS-treated mouse #1	84	356
DSS-treated mouse #2	95	247
DSS-treated mouse #3	68	201
DSS-treated mouse #4	29	109
DSS-treated mouse #5	68	228
DSS-treated mouse #6	67	238
formalin-injected mouse #1	83	408
formalin-injected mouse #2	75	456
formalin-injected mouse #3	52	236
formalin-injected mouse #4	49	358
formalin-injected mouse #5	13	70
formalin-injected mouse #6	10	45
